# From Metabolism to Vitality: Uncovering Riboflavin’s Importance in Poultry Nutrition

**DOI:** 10.3390/ani13223554

**Published:** 2023-11-17

**Authors:** Yauheni Shastak, Wolf Pelletier

**Affiliations:** Nutrition & Health Division, BASF SE, 67063 Ludwigshafen am Rhein, Germany

**Keywords:** riboflavin, vitamin B_2_, supplementation, poultry, redox reactions, oxidative stress, requirements

## Abstract

**Simple Summary:**

Riboflavin, an essential B-vitamin, plays a crucial role in poultry metabolism, impacting energy production, growth, and immune regulation. Its role in redox reactions and energy metabolism is vital for optimal growth and development. Riboflavin is essential for ATP production and the conversion of tryptophan into niacin. Deficiency can lead to skeletal deformities, impaired growth, and compromised immune function. Dietary riboflavin supplementation is necessary due to variable bioavailability in plant-derived sources. The vitamin is absorbed through specialized transport proteins, and its cellular uptake is facilitated by specific receptors. Riboflavin’s role in protein synthesis and its antioxidant properties influence poultry growth and defense against oxidative stress. Its impact on reproductive performance, hatchability, and overall poultry health underscores its significance in poultry nutrition. Future research should focus on its interactions with other nutrients, exploring analogs, and integrating advanced technologies like precision nutrition and nanotechnology for enhanced delivery.

**Abstract:**

Riboflavin, or vitamin B_2_, is indispensable for poultry, profoundly impacting their metabolic equilibrium, growth, and overall health. In a climate of increasing demand for poultry products and heightened production intensity, grasping the multifaceted roles of riboflavin in domestic fowl nutrition becomes paramount. This essential vitamin serves as a precursor to two vital coenzymes, flavin mononucleotide and flavin adenine dinucleotide, integral players in pivotal redox reactions and energy metabolism. Inadequate riboflavin levels translate into stunted growth, skeletal deformities, and compromised feed conversion efficiency, thereby adversely affecting poultry performance and bottom-line profitability. Riboflavin goes beyond its fundamental role, ameliorating nutrient utilization, facilitating protein synthesis, and augmenting enzyme activity, rightfully earning its epithet as the “growth-promoting vitamin”. Poultry’s reproductive success intricately hinges on riboflavin levels, dictating egg production and hatchability. It is imperative to note that riboflavin requirements exhibit variations among poultry species and distinct production phases, emphasizing the importance of judicious and balanced supplementation strategies. Aligning dietary recommendations with genetic advancements holds the promise of fostering sustainable growth within the poultry sector. Exploring the multifaceted aspects of riboflavin empowers researchers, nutritionists, and producers to elevate poultry nutrition and overall well-being, harmonizing with the industry’s evolving demands.

## 1. Introduction

In the ever-evolving landscape of poultry farming, the critical role of nutrition in maximizing productivity and promoting avian health has gained significant attention. Domestic fowl, specifically chickens, turkeys, geese, and ducks, hold a paramount position in the global animal protein industry, meeting the rising demand for affordable and nutritious protein sources. Forecasts indicate that by 2031, approximately 47% of the protein sourced from meat will stem from poultry, overshadowing pig, sheep, and bovine meat consumption [1]. Within the intricate web of micronutrients necessary for poultry well-being, the B-vitamins, a group of water-soluble compounds, play a vital role in various physiological processes [2,3]. Among these, riboflavin, also known as vitamin B_2_, emerges as a pivotal player in maintaining metabolic equilibrium, ensuring optimal growth and safeguarding the health of poultry [4,5,6,7]. The recognized nomenclature by the International Union of Pure and Applied Chemistry (IUPAC) for riboflavin is 6,7-dimethyl-9-(D-ribityl)isoalloxazine.

Riboflavin, standing prominently as one of the eight essential B-vitamins, assumes an unparalleled role due to its involvement in an extensive array of metabolic reactions that constitute the foundation of life [8,9]. Functioning as a critical coenzyme precursor, vitamin B_2_ orchestrates pivotal redox reactions, contributing indispensably to the intricate processes of energy production, growth facilitation, and the coordination of immune responses [10,11]. As modern poultry production systems witness an intensification in their practices and the demand for poultry-derived products continues its upward trajectory, the imperative for an all-encompassing grasp of micronutrient prerequisites amplifies manifold. In this context, riboflavin emerges as a linchpin micronutrient, capable of exerting far-reaching impacts on poultry performance and holistic well-being [12,13].

Central to the matter lies the astounding metabolic demands of poultry, characterized by rapid growth kinetics and inherent production prowess, necessitating substantial nutritional resources [14]. Vitamin B_2_ is a precursor for two important coenzymes: flavin mononucleotide (FMN), involved in the mitochondrial electron system, and flavin adenine dinucleotide (FAD), associated with various proteins in redox reactions [15]. These redox reactions are pivotal for the breakdown of carbohydrates, fats, and proteins, culminating in the production of adenosine triphosphate (ATP), the cell’s primary energy currency [16]. Deficiency in this essential micronutrient could disrupt balanced metabolic functioning in poultry, resulting in delayed growth, skeletal deformities, and worsened feed conversion efficiency, a critical metric in poultry farming [8,17]. Frequent clinical indications encompass limping, paralysis, toes curling inward, and elevated culling rates [17]. As riboflavin insufficiency reverberates through metabolic pathways, its consequences extend even to immunity [18,19].

This escalating complexity propels riboflavin to the forefront of nutritional concerns in the poultry sector. The intricate interplay between this seemingly unremarkable vitamin and the complex pathways of metabolism surpasses mere scientific curiosity, holding direct economic implications for poultry production [13,20,21,22,23,24,25,26]. Riboflavin’s dual role as a conductor of energy dynamics and an architect of growth processes underscores the delicate equilibrium underlying poultry nutrition. By elucidating how vitamin B_2_ shapes metabolic landscapes and influences avian health, nutritionists, researchers, and poultry producers can advance towards enhanced sustainability and efficiency in the industry’s landscape. As the poultry industry continues its ascendancy [27], riboflavin’s centrality as a cornerstone of avian nourishment becomes increasingly pronounced [9]. Table 1 provides a summary of the primary functions of vitamin B_2_.

While plants may contain significant amounts of vitamin B_2_ synthesized through biochemical pathways [34], animal tissues and by-products generally exhibit comparatively lower riboflavin concentrations [35]. The riboflavin content in plant-based feedstuffs can substantially vary due to factors like soil quality, climate, and agricultural practices [36]. Given this variability, supplementing animal diets with riboflavin-containing additives or vitamin mixes becomes crucial for meeting optimal requirements in livestock growth, development, and overall performance. Maintaining a balanced and sufficient vitamin B_2_ intake is vital for bolstering the health of both animals and humans [10].

This review seeks to unravel the multifaceted roles of vitamin B_2_ in metabolism, growth, and health within poultry populations. Our concentration will be on the following key facets:

1. Biochemical Fundamentals of Riboflavin;
2. Riboflavin Metabolism in Poultry;
3. Riboflavin and Poultry Growth;
4. Oxidative Stress Defense;
5. Reproductive Performance and Hatchability;
6. Riboflavin Requirements for Poultry;
7. Future Prospects and Research Avenues.

By comprehending riboflavin’s intricate interplay with poultry physiology, nutritionists, researchers, and poultry producers can collaboratively pave the way for innovative nutritional strategies that sustainably advance the poultry industry.

## 2. Biochemical Fundamentals of Riboflavin

Riboflavin plays a key role in the fundamental biochemical processes that sustain avian life. Its significance arises from its multifaceted functions as a coenzyme in a variety of metabolic reactions, notably those involving redox reactions and energy metabolism [15,26]. To grasp the biochemical underpinnings of riboflavin’s role in poultry health, it is imperative to delve into the intricate details of its molecular structure and its coenzyme derivatives, namely FMN and FAD. Furthermore, comprehending its key functions within the avian system, encompassing its involvement in redox reactions and facilitation of energy metabolism, illuminates its indispensability for optimal growth and development.

**Figure 1:**
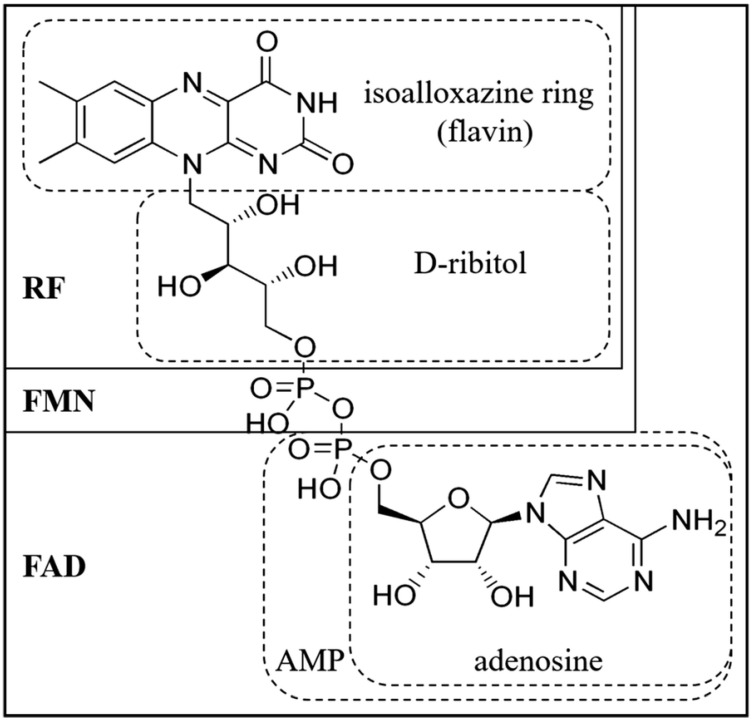
Chemical structure and nomenclature of flavins (Liu et al. [38]). RF = riboflavin; FMN = flavin mononucleotide; FAD = adenine dinucleotide; AMP = adenosine monophosphate.

At its core, riboflavin comprises a heterocyclic ring system, consisting of a central isoalloxazine ring intricately linked to a ribitol side chain [10,37]. This distinctive chemical arrangement enables riboflavin to serve as a precursor to its coenzyme derivatives, FMN and FAD [38] (Figure 1). FMN is synthesized through the phosphorylation of riboflavin catalyzed by riboflavin kinase in the presence of ATP:Mg^2+^. This conversion represents a major rate-limiting step in FAD biosynthesis [39]. FAD formation occurs as FMN:ATP adenylyl transferase catalytically adenylylates FMN to produce FAD [40]. FMN and FAD are tightly associated with enzyme cofactors that can either accept or donate two electrons and two protons to achieve full reduction or a single electron and proton to form the semiquinone intermediate [41]. This coenzyme system facilitates electron transfer during biochemical reactions, establishing riboflavin as an indispensable component in enzyme-catalyzed oxidation reduction processes in avian species as well as mammals [26,42].

Fundamentally, the biochemical functions of riboflavin in poultry are intricately linked to its role as a coenzyme in redox reactions [29]. These reactions play a crucial role in maintaining the delicate equilibrium between the oxidized and reduced states of molecules within the cellular environment. The coenzymes of riboflavin, namely FMN and FAD, play an active role in these reactions by functioning as carriers of electrons [41]. When enzymes facilitate reactions involving electron transfer, the coenzymes FMN and FAD alternate between their oxidized and reduced states, effectively shuttling electrons to and from the reaction site [43,44].

This unique ability to mediate electron transfer is particularly crucial in enzymatic reactions taking place within the mitochondria—the cellular powerhouses. This is where oxidative phosphorylation occurs [45,46]. Given this backdrop, riboflavin’s coenzymes serve a vital role in the electron transport chain, an integral process for generating ATP [28]. In its inherent capacity, riboflavin functions as a cofactor for multiple enzymes, playing a crucial role in the synthesis of prosthetic groups within them. Illustrative examples encompass cytochrome reductase, lipoamide dehydrogenase, xanthine oxidase, L- and D-amino acid oxidase, as well as histaminase [47]. The pivotal function of these enzymes lies in facilitating the indispensable redox reactions that underlie cellular respiration.

**Figure 2:**
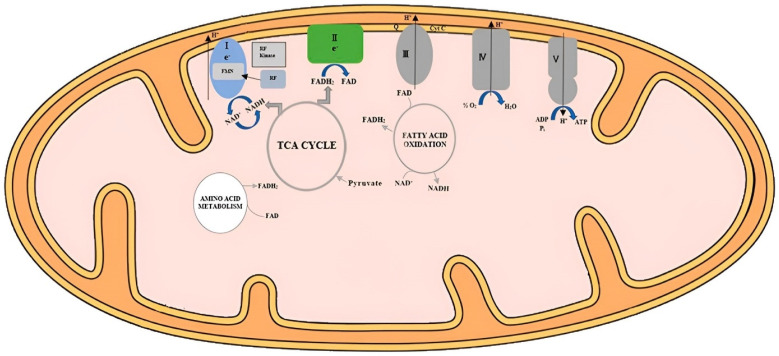
Schematic diagram of flavocoenzymes in mitochondrial energy metabolism (Balasubramaniam et al. [28]). “The OXPHOS system is a 5-enzyme complex which encompasses the mitochondrial respiratory chain (Complexes I-IV), Complex V, and two mobile electron shuttles (coenzyme Q10 and cytochrome c). Electrons derived from oxidation of pyruvate mediated by pyruvate dehydrogenase (PDH) and fatty acid oxidation are transferred via NADH to Complex I (FMN-dependent NADH-ubiquinone oxidoreductase), while electrons from succinate in the Krebs cycle, amino acid metabolism, and fatty acid oxidation are transferred to Complex II (FAD-dependent succinate-ubiquinone oxidoreductase) via FADH_2_. Electrons are subsequently transferred to ubiquinone (Coenzyme Q10) and then to Complex III (reduced CoQ-cytochrome c reductase), and via cytochrome c to cytochrome c oxidase (COX) (Complex IV), the terminal oxidase of the RC before finally reducing molecular oxygen to water. The free energy liberated during this sequential electron transfer is used to generate an electrochemical gradient of protons, which is finally used by Complex V (ATP synthase or F1F0 ATPase) to drive ATP synthesis from ADP and inorganic phosphate. RF: Riboflavin; RF kinase: riboflavin kinase; Q: Coenzyme Q10; CytC: cytochrome c oxidase; FMN: flavin mononucleotide; FAD: flavin adenine dinucleotide”.

In the context of energy metabolism, riboflavin’s significance cannot be overstated. Poultry, like all living organisms, requires energy for various physiological processes, including growth, maintenance, and reproduction. Riboflavin’s involvement in energy metabolism primarily stems from its participation in the electron transport chain [26,28] (Figure 2). During this process, electrons are shuttled through a series of protein complexes, leading to the pumping of protons across the membrane [48,49]. The resulting proton gradient is then used to drive the synthesis of ATP, which serves as the energy reservoir for cellular activities [50].

Moreover, riboflavin’s influence on energy metabolism extends beyond its impact on mitochondria; it assumes a central role in the intricate processing of macronutrients such as carbohydrates, lipids, and proteins [51]. Notably, riboflavin-dependent enzymes referred to as dehydrogenases actively participate in the oxidation of glucose and fatty acids [52,53]. This oxidative process stands as a pivotal stage in harnessing energy from these substrates. Furthermore, riboflavin is a key player in the conversion of the amino acid tryptophan into niacin, an essential B-vitamin [54,55]. Although the efficiency of this conversion, as illustrated in turkey poults, may be limited, it nevertheless contributes to the provision of niacin—an indispensable element for diverse metabolic mechanisms in poultry [54].

In the domain of poultry well-being, insufficiency in vitamin B_2_ can yield deleterious repercussions [6,8]. Avians lacking in riboflavin may manifest various clinical indications, encompassing diminished growth rates, musculoskeletal abnormalities, peripheral nervous system impairment, and compromised reproductive capabilities [4,12,17,56]. These manifestations trace their origins to the disruption of energy metabolism and redox reactions, both of which are indispensable for sustaining the heightened metabolic requisites of fast-growing poultry [57]. Riboflavin deficiency can lead to a curtailed capacity in ATP generation, culminating in reduced energy availability for growth and maintenance [7]. Furthermore, the disruption of redox reactions can compromise the cell’s proficiency in fending off oxidative stress, consequently precipitating cellular damage and compromised immune functionality [58].

## 3. Riboflavin Metabolism in Poultry

Understanding the metabolism of riboflavin in domestic fowl is vital for ensuring optimal health, growth, and production in these birds. The journey of vitamin B_2_ in poultry begins with its absorption, transport, and subsequent tissue distribution.

The absorption mechanism of riboflavin predominantly takes place within the small intestine of domestic fowl [59]. This intricate process commences with the liberation of riboflavin from dietary sources. Common constituents of poultry diets, such as grains and protein-rich meals, contain varying concentrations of vitamin B_2_ [60,61,62]. As elucidated by Merrill et al. [63], the major portion of riboflavin in feed materials exists in the form of free coenzymes—FMN and FAD—the predominant being FAD. The availability of riboflavin to the avian system necessitates its prior release from these coenzymes, which is facilitated by gut pyrophosphatases and phosphatases [64]. This step is crucial for the subsequent absorption of riboflavin.

Due to the inherent variability in the vitamin B_2_ content of plant-derived ingredients, coupled with factors leading to variable bioavailability and occasional degradation, the supplementation of fermentation-synthesized riboflavin via premixes becomes essential to fulfill the vitamin’s requirements in animal nutrition [35,36,64,65,66]. Industrially produced riboflavin, being in a non-esterified form, can be directly absorbed without the need for a hydrolysis step, which is necessary for plant-derived native vitamin B_2_. However, even in this case, the liberation of riboflavin from the feed matrix post-ingestion remains a prerequisite, achieved through mechanical breakdown and enzymatic activity along the avian digestive tract [67]. This liberation phase precedes absorption since riboflavin must be in its unbound form to be effectively taken up by the system [64].

Following liberation from feed particles, riboflavin emerges into an aqueous environment within the digestive tract, undergoing solubilization to facilitate subsequent absorption. The loss of biosynthetic pathways for most vitamins in the ancestors of vertebrates led to the development of mechanisms such as specialized transport proteins [68]. These proteins aid in the uptake of dietary vitamins from both the intestine and serum [69]. In the case of vitamin B_2_, a crucial step in its intestinal absorption involves active transportation across epithelial cells via specialized riboflavin transporters, ensuring the efficient flux of the vitamin into the bloodstream [19]. Under physiological concentrations, riboflavin is taken up through an active and saturable transport mechanism [70]. Although Cordona and Payne’s [59] study did not discern significant differences in riboflavin absorption across various segments of the small intestine in chickens, it is conceivable that vitamin B_2_ is predominantly absorbed in the proximal section of the small intestine, similar to the absorption pattern observed for other vitamins [71]. This proposition finds support in human studies, which indicate that riboflavin is primarily absorbed in the proximal small intestine [72].

Vitamin B_2_ transportation within poultry species is facilitated by its association with specialized transport proteins, ensuring its equitable dispersion throughout the avian organism [73,74]. In these species, riboflavin is conveyed via the circulatory system, serving as the conduit for its movement. An instrumental component in this mechanism is the chicken riboflavin-binding protein (RBP), a phosphoglycoprotein weighing 29.4 kDa [75,76,77]. The pivotal role of RBPs lies in safeguarding riboflavin from degradation, facilitating its secure transfer to diverse tissues [69]. By means of the RBP-mediated process, a consistent supply of vitamin B_2_ is assured to organs and tissues reliant on this vital micronutrient for optimal functioning.

**Figure 3:**
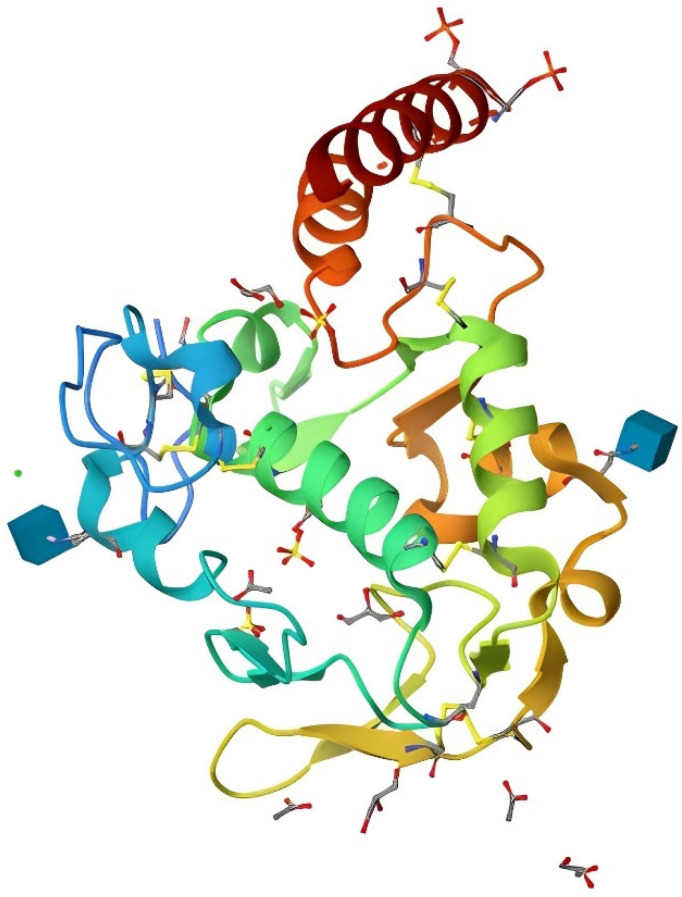
Crystal structure of chicken riboflavin-binding protein in “Apo” form at 2.5 A resolution (Loch et al. [77]).

The gene responsible for producing egg white, yolk, and serum RBPs is shared, exhibiting slight tissue-specific disparities in post-translational modifications [68]. RBPs are synthesized by the liver or oviduct in poultry and subsequently released into the bloodstream or eggs [26,78]. The mature and functional form of RBP (depicted in Figure 3) undergoes initial post-translational modifications [73]. This entails cleavage of an unidentified signal peptide, blockade of its amino terminus with pyroglutamic acid, and excision of an 11-13-residue acidic carboxyl-terminal peptide during or after transportation [79]. Additionally, nine disulfide bonds have been identified, along with two N-linked oligosaccharides whose composition varies based on the synthesizing tissue, and a serine-rich region bearing eight phosphoryl groups [73,80,81]. Notably, these phosphoryl groups play a critical role in facilitating the transportation of serum RBP into diverse tissues [75].

The cellular uptake process of riboflavin involves the transport of the vitamin across the plasma membrane via specific receptors called riboflavin transporters, following its release from the plasma through the RBP [78]. This mechanism relies on a calcium-ion-dependent RBP receptor, situated within clathrin-coated pits on the phospholipid bilayer [82]. This receptor serves as a catalyst for the endocytosis of the vitamin B_2_, enabling its subsequent internalization and release [78]. Subsequently, the receptor and RBP undergo recycling, while catabolic processes occur within endosomes.

The distribution of vitamin B_2_ in various tissues provides valuable insights into its significance in avian metabolism, particularly its involvement in enzymatic reactions. Riboflavin plays a critical role as a precursor to two essential coenzyme forms: FMN and FAD [38]. Tissues with elevated energy demands, such as muscle and liver, exhibit higher concentrations of riboflavin due to their reliance on FMN and FAD [83,84,85]. Moreover, tissues engaged in redox reactions, including the heart and kidney, maintain substantial riboflavin levels due to the participation of FAD-dependent enzymes in these processes [70,72]. When an abundant quantity of riboflavin is absorbed by the small intestine, surpassing the body’s current needs, the excess riboflavin is efficiently eliminated from the bloodstream and excreted through urine without undergoing significant alteration [86]. Nonetheless, a portion of vitamin B_2_ is excreted through the renal route in the form of metabolites. These metabolites result from oxidative cleavage in the ribityl side chain and subsequent conversion of the ring methyl functions to hydroxymethyl groups [87].

The enzymatic pathways responsible for the conversion of riboflavin into its active coenzyme forms, FMN and FAD, represent a pivotal facet of riboflavin metabolism in poultry. These conversions occur through a series of enzymatic reactions collectively referred to as the riboflavin kinase pathway [88]. The initial step involves the phosphorylation of riboflavin, catalyzed by riboflavin kinase, resulting in the formation of riboflavin 5′-phosphate [42,89]. This phosphorylation step is crucial for the subsequent transformations. The final conversion entails the synthesis of FAD from FMN (i.e., riboflavin 5′-phosphate) and ATP, facilitated by FAD synthetase [78]. These coenzyme derivatives, FMN and FAD, become indispensable participants in a range of redox reactions, spanning from the electron transport chain to various dehydrogenase reactions [29,41]. 

## 4. Riboflavin and Poultry Growth

An important observation from the first half of the 20th century is that riboflavin was designated as the “growth-promoting vitamin G” [90,91,92,93,94,95]. This recognition is rooted in riboflavin’s involvement in a multitude of physiological processes intricately linked to the overall growth performance of domestic fowl species. Within this complex web of biological interactions, vitamin B_2_ significantly influences pivotal factors that contribute to poultry growth, including nutrient utilization, protein synthesis, and enzyme activity [7,30,93,96].

The interrelation between vitamin B_2_ and nutrient utilization constitutes a fundamental aspect in comprehending its influence on poultry growth. Riboflavin serves as a cofactor for enzymes engaged in the metabolic pathways responsible for the degradation of carbohydrates, lipids, and proteins [10,97]. Through facilitation of these enzymatic reactions, riboflavin indirectly enhances the efficacy of nutrient breakdown and absorption within the avian gastrointestinal tract [26]. This, in turn, culminates in a heightened extraction of energy and nutrients from the ingested feed, thereby furnishing the essential foundational components required for optimal growth. The amelioration in nutrient utilization serves as a principal catalyst underpinning the growth-promoting ramifications of riboflavin across diverse poultry species [25,30,91,98].

Chou et al. [98] initially underscored that a reduction in energy or protein intake possesses the potential to curtail the riboflavin necessity in juvenile chickens. Significantly, their investigation unveiled that a marked deficiency of riboflavin (2.26 mg/kg diet) elicited a substantial regression (*p* < 0.01) in both energy and protein utilization among ad libitum-fed chicks. Analogously, a marginal insufficiency of riboflavin (3.02 mg/kg diet) was found to diminish energy utilization (*p* < 0.01) in chicks subjected to unrestricted feeding. Furthermore, the severe dearth of riboflavin was noted to engender diminished protein utilization (*p* < 0.05) in chicks exposed to energy-restricted diets (ranging from 80% to 60% of controls).

The Tricarboxylic Acid cycle (TCA) holds pivotal significance in the growth of living organisms, as it induces energy production, furnishes foundational constituents, and upholds redox equilibrium [99]. FAD and FADH_2_, integral to the TCA cycle, assume a pivotal role by engaging in redox reactions that contribute to energy synthesis and streamlined growth processes [100]. Dysfunction within this cycle or disruptions in FAD/FADH_2_ participation can impede growth-associated pathways and cellular functionality.

Shifting focus beyond nutrient utilization, the significance of riboflavin in protein synthesis emerges as a pivotal factor in promoting poultry growth. The process of protein biosynthesis, fundamental to the development of muscles, tissues, and bodily structures in both mammals and avian species, involves riboflavin. Specifically, vitamin B_2_ plays a role in the folding of newly synthesized proteins within the endoplasmic reticulum, facilitated by an FAD-dependent enzyme called endoplasmic reticulum oxidoreductase 1 [101,102]. Hypovitaminosis B_2_ can potentially disrupt this protein folding process due to diminished flavoproteins and an imbalanced redox state, triggering a stress response within the endoplasmic reticulum [102]. Research has also indicated that insufficiency of vitamin B_2_ significantly reduces glutathione reductase activity and glutathione content, while downregulating the expressions of endoplasmic reticulum oxidoreductase 1 and protein disulfide isomerase, as observed in poultry and other species [12,26,103,104,105].

Empirical evidence supporting the influence of riboflavin on domestic fowl growth is substantial and compelling. Diverse experiments across various poultry species have been conducted to explore the impact of vitamin B_2_ supplementation on growth performance [4,5,8,13,17,25,33,90,91,92,93,94,95,98]. For example, in broiler chickens, the consistent outcome of dietary riboflavin supplementation has been linked to improvements in growth rate, feed conversion efficiency, and carcass yield [24,60,106]. These growth-promoting effects are attributed to riboflavin’s contributions to nutrient utilization, as its involvement in metabolism clearly correlates with improved growth metrics.

Likewise, investigations centered on turkey poults have substantiated the favorable impact of vitamin B_2_ on their growth and developmental processes [61,107,108]. Supplementation of riboflavin in the diets of turkeys, ducks, and chickens has also been linked to enhanced feather development. Moreover, insufficient levels of vitamin B_2_ have been associated with conditions like the “clubbed down syndrome” or the twisting of feather follicles, alongside instances of dermatitis [26,109,110,111,112]. The “clubbed down syndrome” refers to the failure of down feather follicles to rupture their surrounding sheaths, causing feathers to coil and take on the appearance of a French knot [113]. This link underscores the interrelation between riboflavin and protein synthesis, as the synthesis of specialized proteins is crucial for proper feather and skin development [114]. Riboflavin’s role in providing essential coenzymes for protein synthesis and proper folding contributes significantly to the production of these proteins, consequently fostering feather growth [102,115].

The growth of ducklings also offers a pertinent illustration of riboflavin’s influence on growth. Analogous to observations in chickens and turkeys, studies conducted on ducks have indicated that a deficiency in vitamin B_2_ leads to diminished growth rates and compromised overall health [116]. Researchers in the study assessed specific proteins in the liver through Western blotting techniques. The reduced presence of certain proteins in the tissue was primarily associated with fatty acid β-oxidation and the mitochondrial electron transport chain (ETC), implying that hypovitaminosis B_2_ may contribute to liver lipid accumulation and growth impediment by hampering fatty acid β-oxidation and the ETC process. Table 2 summarizes the impact of riboflavin supplementation on the performance of poultry.

## 5. Oxidative Stress Defense

Oxidative stress, characterized by an imbalance between the production of reactive oxygen species (ROS) and cellular detoxification mechanisms, presents a significant challenge to domestic fowl health and productivity [58]. Avian cells have developed an intricate defense network to counter this threat, with riboflavin emerging as a pivotal component in this system [11]. Recent investigations have unveiled an additional dimension to riboflavin’s role—its function as a potent antioxidant within animal cells [30]. This discovery underscores its critical contribution to ROS neutralization and cellular protection, rendering it indispensable in poultry’s defense against redox imbalance [26].

The complex metabolic processes inherent to avian cells inevitably lead to the generation of ROS as natural by-products. While ROS serve important physiological roles, their excessive accumulation triggers oxidative stress, inducing damage to lipids, proteins, and nucleic acids [119]. Such cellular damage disrupts vital functions, compromises immune responses, and fosters the onset of diverse poultry ailments [58]. Herein lies the significance of riboflavin’s role as an antioxidant. Riboflavin partakes in various enzymatic reactions, particularly within the mitochondria’s electron transport chain [26,28], aiding in nutrient conversion for energy production. Yet, its fundamental antioxidant capabilities stem from its role as a precursor for two crucial coenzymes, FMN and FAD. These coenzymes play a central role in the activity of key antioxidant enzymes, including glutathione reductase and lactate dehydrogenase, indispensable for cellular defense against oxidative stress in animal cells, including domestic fowl [23,30,120,121].

**Figure 4:**
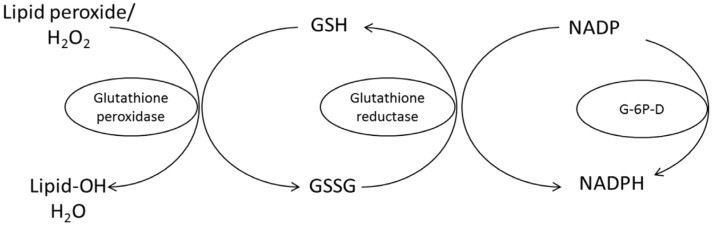
Conversion of oxidized glutathione (GSSG) to the reduced form (GSH) by glutathione reductase requires riboflavin in the flavin adenine dinucleotide (FAD) coenzyme form for its activity (Suwannasom et al. [10]). G-6P-D = glucose-6-phosphate dehydrogenase.

Central to riboflavin’s role as an antioxidant is its active involvement in the glutathione redox cycle. Glutathione, a robust tripeptide antioxidant, plays a pivotal role in defending against damage induced by ROS. Vitamin B_2_ contributes to this cycle by facilitating the activity of glutathione reductase. This enzymatic action promotes the regeneration of reduced glutathione from its oxidized state, as detailed by Suwannasom et al. [10] (Figure 4). This dynamic process is crucial for maintaining cellular redox equilibrium, a fundamental factor in mitigating oxidative stress.

Zhang et al. [11] conducted a study centered around White Pekin duck breeders to investigate the impact of varying dietary riboflavin levels (ranging from 0 to 15 mg/kg) on oxidative status. The results demonstrated that duck breeders deprived of vitamin B_2_ supplementation experienced a significant decrease in their antioxidant capacity. This was evident from elevated plasma malondialdehyde levels and diminished glutathione levels. These findings underscore the pivotal role of riboflavin in fortifying the oxidative defense mechanisms of duck breeders via the glutathione pathway.

Furthermore, in vitro experiments revealed that an oxidized form of riboflavin engages in redox reactions with organic radicals and superoxide anions. This interaction results in the formation of a leuko form, which can be further oxidized by oxygen or iron-containing proteins [122]. As a consequence, riboflavin generates hydrogen peroxide or ferro forms of heme-containing proteins, underlining its potential as an antioxidant. Its interplay with superoxide anions may also contribute to stabilizing nitric oxide levels, further highlighting its significance in mitigating oxidative stress and sustaining cellular well-being [122].

The significance of riboflavin’s robust antioxidant capabilities becomes notably more conspicuous when contextualized within the contemporary landscape of poultry farming. Modern techniques of poultry production expose avian species to an assortment of stress-inducing factors, encompassing elevated temperatures, pathogenic microorganisms, and environmental pollutants. These factors collectively contribute to an exacerbation of oxidative stress conditions [58,123]. Moreover, the deliberate genetic selection aimed at promoting accelerated growth rates and heightened egg production imposes supplementary metabolic burdens on the avian cells, potentially leading to an amplification of the production of ROS [124].

**Figure 5:**
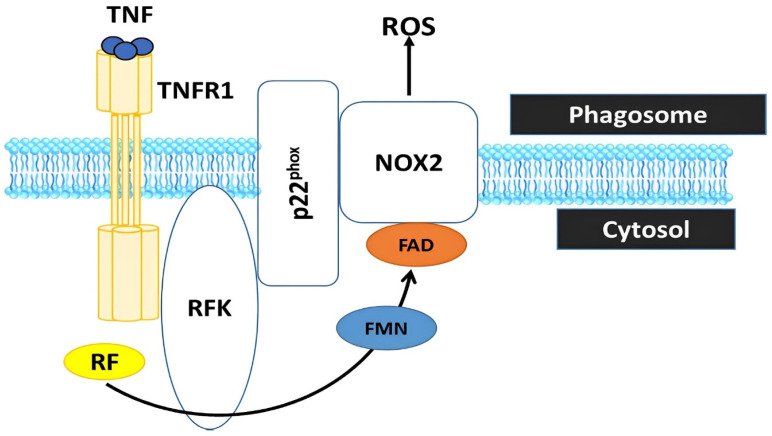
Riboflavin is converted by riboflavin kinase into flavin monophosphate (FMN) and flavin adenine dinucleotide (FAD), which are essential cofactors of the phagocytic NADPH oxidase 2 (Nox2) to generate reactive oxygen species (ROS). Riboflavin deficiency renders the phagocyte Nox2 incapable of producing ROS, a process crucial for deactivating phagocytosed microbes and regulating the inflammatory response in innate immune cells (Suwannasom et al. [10]). TNF = tumor necrosis factor; TNFR1 = tumor necrosis factor receptor 1.

In such intricate scenarios, the availability of riboflavin and other vital antioxidant vitamins, including retinol, α-tocopherol, L-ascorbic acid, and calciferol, assumes utmost importance [58,125]. The introduction of riboflavin through dietary supplementation has demonstrated its capacity to fortify the poultry’s antioxidant defense mechanisms, thereby engendering an ameliorated state of overall health and performance [11,23,120,121]. The beneficial impacts extend beyond a mere enhancement in antioxidant potential and growth facilitation; riboflavin supplementation additionally exerts a constructive influence on immune functions [126] (Figure 5), culminating in an elevated state of avian well-being and concurrently fostering favorable economic outcomes [7,9,13].

## 6. Reproductive Performance and Hatchability

Reproductive performance and hatchability constitute pivotal determinants within the poultry industry, wielding substantial influence over egg production efficiency and the overall triumph of poultry farming [127]. One elemental factor that has been extensively scrutinized in relation to these parameters is riboflavin. The impact of vitamin B_2_ on reproductive variables such as egg production, egg quality, and hatchability has elicited considerable attention from both researchers and poultry producers [11,12,26,56,109,118,128,129,130]. Through an array of studies, it has been unequivocally demonstrated that a dearth of riboflavin can exert a substantial impact on poultry reproductive success, carrying profound ramifications for both economic and bird welfare considerations.

A comprehensive exploration of the ramifications of vitamin B_2_ on reproductive parameters reveals its integral role in sustaining optimal egg production rate [26,56,128]. Riboflavin’s involvement in energy metabolism and cellular function is directly tethered to the energy-intensive process of egg production [11]. A sufficiency of vitamin B_2_ is imperative for supporting the heightened metabolic activity requisite during follicle development and yolk formation [111,118,131]. Research has substantiated that supplementation of riboflavin can culminate in improved egg production rates, yielding a heightened output of superior-quality eggs [118].

Hatchability, a critical parameter in poultry farming, is significantly influenced by riboflavin status [132,133,134,135]. In fact, hypovitaminosis B_2_ emerges as one of the prevalent nutritional insufficiencies capable of influencing the hatching process [136]. The various developmental stages of an embryo within an egg demand a consistent supply of nutrients and energy. Riboflavin’s role in cellular energy generation through its participation in the electron transport chain bears a direct connection to embryonic development and viability [137,138]. A lack of riboflavin during incubation can lead to impaired growth and development of the embryo, ultimately resulting in reduced hatchability rates [139]. Furthermore, the antioxidant properties of vitamin B_2_ play a pivotal role in safeguarding the nascent embryo from oxidative stress, a factor with potential to imperil embryonic viability [12,26,140,141].

The mechanisms through which riboflavin deficiency might impact reproductive success are multifaceted and involve intricate physiological processes. One primary mechanism is tied to riboflavin’s role in energy metabolism, where hypovitaminosis B_2_ can lead to a metabolic crisis [26]. As previously mentioned, riboflavin, in its coenzyme forms FMN and FAD, participates in oxidative phosphorylation, a process critical for ATP production, the primary energy currency of cells [28]. Inadequate vitamin B_2_ levels could lead to impaired energy production, affecting the high energy demands of reproductive processes such as follicle development, egg formation, ovulation, and embryo development and viability [128,139,142]. Hatchability is primarily affected by hypovitaminosis B_2_, followed by a decrease in egg production [143]. Additionally, evidence suggests a direct correlation between the amount of riboflavin in the hen’s diet and the vigor and viability of the baby chick [144].

Moreover, riboflavin’s impact on reproductive success can also be attributed to its involvement in cellular growth and differentiation. Vitamin B_2_ plays a crucial role in maintaining the integrity of cell membranes through its participation in lipid metabolism and the regulation of oxidative stress [11,102,145]. Riboflavin deficiency can disrupt cellular membrane structure and function, affecting the development and viability of reproductive cells [145]. This disruption may potentially yield impaired follicle development, disrupted ovulation, and compromised sperm and oocyte quality, ultimately influencing hatchability rates. The summary in Table 3 presents the effects of riboflavin supplementation on poultry reproductive performance and hatchability.

## 7. Riboflavin Requirements for Poultry

The recommended dietary requirements for vitamin B_2_ vary among different poultry species and at different stages of their production cycle, reflecting the dynamic nature of avian growth and development. In broiler chickens, for instance, riboflavin needs are influenced by their rapid growth during the fattening phase [25]. This phase, characterized by rapid muscle and skeletal development, necessitates increased riboflavin intake, along with other essential vitamins, to support energy metabolism and tissue repair [148]. Conversely, when considering laying hens during their peak egg production phase, distinct vitamin B_2_ requirements emerge [11,56]. Turkeys, with their unique growth characteristics and reproductive patterns, differ from both meat-type and laying-type chickens, resulting in varying nutritional needs, including specific considerations for riboflavin, when compared to broilers and layers (Table 4).

Beyond species and production stages, riboflavin requirements are intricately tied to feed composition [13]. The delicate interplay between vitamin B_2_ and other nutrients underscores the importance of a balanced diet [64]. Poor-quality feeds can hinder riboflavin absorption and utilization, leading to deficiencies despite adequate dietary levels. Additionally, stressors, drug usage, and disease challenges amplify avian metabolic demands, prompting heightened vitamin requirements [35]. In stressful conditions, be they environmental, physiological, or pathogenic, riboflavin’s role in antioxidant defense systems becomes increasingly vital, aiding in the preservation of cellular integrity and resilience in adverse conditions [165].

It is crucial to distinguish between vitamin B_2_ requirement estimates and allowances as determined by scientific committees such as the National Academies of Sciences, Engineering, and Medicine [149] (formerly known as the National Research Council), the Gesellschaft für Ernährungsphysiologie (GfE) [150], and recommendations from poultry breeding companies like Aviagen, Cobb-Vantress, and Lohmann [153,154,155,158]. Poultry producers often refer to both sources to strike a balance between scientific knowledge and the specific genetic potential of their flocks. However, there is a pressing need for an update of the NASEM requirement estimates, given that their last revision dates back to 1994, and substantial genetic advancements have been made in broilers, turkeys, and laying hens.

In contemporary times, there is a growing acknowledgment that the vitamin requirements for commercial poultry production may surpass the previously established levels for healthy birds in controlled research settings, as outlined by organizations like NASEM [71]. Stress, infections, and illnesses can substantially elevate the vitamin needs of birds, factors that must be taken into account in real-world farming scenarios [166]. For example, the most recent recommendations from Aviagen and Cobb [153,154], dated 2022, are designed to ensure adequate vitamin levels in commercial farming operations, probably factoring in potential vitamin losses during storage and processing by incorporating a safety margin (see Table 4).

Understanding the complex roles of riboflavin requires a comprehensive perspective. From an energy standpoint, vitamin B_2_ plays a pivotal role in metabolic pathways that extract energy from nutrients [100]. By facilitating the conversion of carbohydrates, fats, and proteins into usable energy units, riboflavin contributes to poultry’s overall vigor and vitality [26,28]. This function is especially critical during the brooding period, as young chicks transition from yolk-derived sustenance to external feed sources. Special attention to breeder requirements for vitamin B_2_ is necessary. Riboflavin’s role as a coenzyme in key enzymatic reactions orchestrates this metabolic shift, ensuring efficient nutrient utilization and sustained growth [38].

Riboflavin’s involvement in cellular growth and repair mechanisms underscores its significance during the rapid growth phase of poultry. Skeletal development, a complex process of bone formation and remodeling, relies heavily on vitamin B_2_-mediated energy transactions. As chicks develop their skeletal framework at a rapid pace, the mineralization process depends on the energy generated through riboflavin-supported metabolic pathways [167]. This not only influences bone strength but also contributes to the overall structural robustness of growing birds. Hypovitaminosis B_2_ can lead to an increase in deformed legs and poor mobility in broilers [117]. Citrate, which comprises approximately 1.6% of bone content and about 80% of total body citrate residing in bones, plays a crucial role in bone stability, strength, and resistance to fracture [168]. Riboflavin in its coenzyme form FAD is critical for the normal functioning of the citric acid cycle, which is presumed to be a key supplier of citrate for the bone’s apatite nanocrystal structure [168]. This occurs through the prevention of citrate oxidation via the Krebs cycle in some bone cells, maximizing citrate accumulation [168].

However, the intricate tapestry of riboflavin’s functions is interwoven with feed composition. Common feed ingredients like corn, wheat, soybean meal, and oilseed meals can contribute to riboflavin content [36]. Nevertheless, the intrinsic levels of vitamin B_2_ are insufficient to meet the requirements of poultry on a commercial scale in most cases [17,24,25,169]. Therefore, premixes used in poultry feeding are universally supplemented with vitamin B_2_ and other vitamins. Yet, inconsistencies or imbalances in premix and feed formulation can potentially lead to riboflavin and other vitamin B group deficiencies [12,170,171]. This underscores the importance of quality control in premix and feed production and the judicious selection of feed ingredients to ensure optimal riboflavin provision.

## 8. Future Prospects and Research Avenues

As we venture into the future of riboflavin research in poultry, it becomes evident that there is a wealth of untapped potential waiting to be explored. Among the most promising directions for research in this field is the investigation of how vitamin B_2_ interacts with other essential nutrients, such as vitamins, minerals, and amino acids. Understanding these interactions can yield valuable insights into optimizing poultry diets for improved growth and performance. For instance, delving into the synergy between riboflavin and other B vitamins could lead to more efficient nutrient utilization, ultimately benefiting poultry health and productivity. It is well-documented in mammalian studies that riboflavin plays a role in the metabolism of other B-vitamins, including vitamin B_3_ (niacin), vitamin B_6_ (pyridoxine), and vitamin B_9_ (folate) [172]. It facilitates the conversion of these vitamins into their active forms, enabling them to fulfill their respective functions in the body. While the impact of riboflavin on niacin synthesis has been demonstrated in turkey poults [54], further research in this area is warranted in domestic fowl.

Another intriguing avenue for exploration in avian health lies in the examination of riboflavin analogs [173]. These analogs have shown promise in offering anti-infective benefits in poultry, as demonstrated in both in vivo and in vitro studies in mammalian and bacterial cells [174]. Investigating this area could shed light on novel compounds that may be incorporated into poultry diets to achieve improved outcomes, including their potential as anticoccidial, antibacterial, and antiviral agents [175,176]. Several studies have already illustrated the therapeutic efficacy of photoactivated riboflavin against nosocomial infections, multidrug-resistant bacterial infections, and microbial-associated biofilm infections. This highlights the potential of riboflavin as a promising antimicrobial candidate, which could play a role in addressing the global crisis of emerging antimicrobial resistance among various pathogenic microbes [177].

In addition to studying riboflavin’s interactions with other nutrients and exploring analogs, integrating advanced technologies holds significant promise for enhancing vitamin B_2_ delivery in poultry diets. One such technology is precision nutrition, which involves customizing poultry diets based on individual bird characteristics and daily nutritional requirements [178]. By leveraging techniques like artificial intelligence and machine learning, researchers can develop predictive models that optimize riboflavin and other vitamin supplementation, potentially tailoring diets for each bird in a flock [179]. This approach can reduce waste, lower production costs, and enhance overall flock performance.

Furthermore, the utilization of nanotechnology in riboflavin delivery to poultry presents another exciting avenue for future research. Nanoencapsulation of riboflavin can enhance its stability and bioavailability, ensuring a consistent and effective supply of this essential micronutrient to poultry [180]. Nanoparticles can protect riboflavin from degradation in the proximal part of the gastrointestinal tract, facilitating more efficient intestinal absorption [181].

Moreover, exploring the potential role of vitamin B_2_ in mitigating the adverse effects of stressors on poultry health is a compelling area of research. Stressors such as heat stress, disease outbreaks, and transportation can negatively impact poultry well-being and productivity [182]. Investigating how riboflavin supplementation can alleviate the detrimental effects of these stressors is a critical research avenue, as already evaluated in humans [183,184]. Understanding the mechanisms by which riboflavin and other vitamins contribute to stress resilience in poultry can lead to innovative solutions for maintaining poultry health and welfare under challenging conditions.

## 9. Conclusions

Riboflavin plays a crucial role in the field of poultry nutrition and health. Its diverse functions as a coenzyme in various metabolic reactions, particularly in redox reactions and energy metabolism, emphasize its indispensability in avian physiology. Vitamin B_2_ significantly contributes to enhancing nutrient utilization, facilitating protein synthesis and folding, and promoting enzyme activity. These roles collectively support optimal growth and performance in domestic fowl. Moreover, the impact of riboflavin on reproductive parameters, such as egg production, egg quality, and hatchability, cannot be overstated. Its involvement in energy metabolism and antioxidant defense mechanisms directly influences the reproductive success of avian species, with far-reaching implications for both economic viability and animal welfare considerations.

Exploring riboflavin’s interactions with other essential nutrients, investigating the potential of riboflavin analogs as antimicrobial agents, and embracing advanced technologies like precision nutrition and nanotechnology for improved riboflavin delivery represent promising avenues for future research in poultry nutrition.

In essence, vitamin B_2_ emerges as a pivotal micronutrient within the intricate web of poultry nutrition, exerting profound effects on growth, health, and reproductive performance. As the poultry industry continues to advance, a deeper understanding of riboflavin’s roles and innovative approaches to its supplementation will prove essential in sustaining and furthering this vital sector.

## Figures and Tables

**Table 1:** Functions of riboflavin.

Function	Description	Reference
Redox reactions and energy production	Riboflavin is essential for producing energy via two key coenzymes, flavin mononucleotide (FMN) and flavin adenine dinucleotide (FAD).	[28,29]
Antioxidant capacity	Riboflavin possesses indirect antioxidant properties, aiding in the neutralization of harmful free radicals within the body.	[11,30,31]
Metabolism of fats, drugs, and steroids	Riboflavin participates in the enzymatic reactions associated with the metabolism of lipids, xenobiotic substances, and steroid compounds.	[32]
Cellular function, growth, and development	Riboflavin plays a fundamental role in the regulation of cellular functions, growth, and developmental processes.	[26]
Reproductive functions	Riboflavin is essential for the reproductive performance of poultry. It affects fertility, embryonic development, and hatchability.	[11,26]
Nerve function	Riboflavin deficiency has been associated with peripheral nerve demyelination in poultry, resulting in symptoms such as leg weakness and curled toe paralysis.	[5,33]

**Table 2:** Impact of riboflavin supplementation on the performance and leg abnormalities of poultry.

Poultry Species	Dietary Supplemental Riboflavin Levels in Feed	Effects of Riboflavin Supplementation on Performance and Leg Abnormalities	Reference
Broiler chicken	0.0, 0.9, 2.0, 2.8, 3.6, 4.4 mg/kg	Improved body weight, feed intake, FCR, and reduced occurrence of leg paralysis	[60]
Broiler chicken	2.75, 2.78, 3.05, 3.40, 3.71 mg/kg	Improved body weight, feed intake, FCR, and reduced occurrence of curled-toe paralysis	[98]
Turkey poults	0.0, 0.6, 1.1, 1.7, 3.1, 4.4 mg/kg	Improved body weight, feed intake, FCR, and reduced occurrence of leg paralysis	[61]
Broiler chicken	1.7, 3.7, 11.7 mg/kg	Bilateral leg weakness and rotation of the metatarsus with flexion of the digits and hock lesions in “the 1.7 mg/kg group” as well as leg weakness in “the 3.7 mg/kg group” compared to the “11.7 mg/kg group”.	[5]
Broiler chicken	1.0–5.0 mg/kg	Improved body weight and FCR	[25]
Broiler chicken	4.0-10.4 mg/kg	Improved daily weight gain, FCR, and European broiler index ^1^	[106]
Pekin ducks	0.0 and 10.0 mg/kg	Lower mortality, improved average daily gain, feed intake, and gain/feed ratio	[116]
Broiler chicken	0.0, 1.0, 2.0, 3.0, 4.0, 8.0 mg/kg	Lower mortality, improved body weight, feed intake, FCR, and reduced occurrence of leg paralysis	[24]
Broiler chicken	0.0, 0.2, 0.5, 0.9, 4.5 mg/kg	Lower mortality, improved body weight, and reduced occurrence of leg paralysis	[21]
Broiler chicken	0.0, 9.0 mg/kg	Improved weight gain and FCR and reduced occurrence of leg paralysis	[117]
Bobwhite quail	0.0, 0.8, 1.5, 2.5, 3.5, 5.0 mg/kg	Lower mortality, improved body weight and FCR	[22]
Ringnecked pheasants	0.0, 0.4, 0.9, 1.3, 1.8, 2.4 mg/kg	Improved weight gain and reduced occurrence of leg abnormalities	[20]
Broiler chicken	0.8, 6.6, 20.0 mg/kg	Improved FCR	[29]
Laying hen	0.0 and 2.9 mg/kg	Improved egg weight	[56]
Broiler breeders	2.5 and 4.0 mg/kg	No effect	[7]
Broiler chicken	2.5 and 4.0 mg/kg	Improved growth rate and feed consumption	[7]
Turkey poults	0.0, 2.0, 4.0 or 8.0 mg/kg	Higher body weight	[23]
Laying hen	1.55, 2.20, 4.40, and 8.80 mg/kg	Improved egg production and egg weight	[118]

^1^ European broiler index = daily weight gain (g) × survival rate (%)/feed conversion (kg feed/kg body weight gain) × 10.

**Table 3:** Impact of riboflavin supplementation on poultry reproductive performance and hatchability.

Poultry Species	Dietary Supplemental Riboflavin Levels in Feed	Effects of Riboflavin Supplementation on Reproductive Performance and Hatchability	Reference
Laying hen	0.0 and 2.9 mg/kg	Improved hatchability	[56]
Laying hen	1.55, 2.20, 4.40, and 8.80 mg/kg	Improved egg production, egg weight, hatchability, and hen weight as well as reduced incidence of hemorrhagic embryos and clubbed down	[118]
Duck breeder	0.0 and 10.0 mg/kg	Improved hatchability	[12]
Duck breeder	0.0 and 16.5 mg/kg	Improved hatchability and embryo weight	[145]
Broiler breeders	2.5 and 4.0 mg/kg	No effect	[7]
Duck breeder	0, 2.5, 5, 10, and 15 mg/kg	Improved hatchability	[11]
Laying hen	0.9–8.1 mg/kg	Improved egg production and hatchability	[146]
White leghorn and Rhode island red breeder hens	1.0 and 2.5 mg/kg	Reduced embryo mortality and number of malpositioned embryos	[147]

**Table 4:** Vitamin B2 guidelines for poultry: requirement estimates (NASEM), allowances (GfE), and recommendations (remaining sources).

Source	Vitamin A Requirement, mg/kg Feed			
	Broilers	Laying Hens	Broiler Breeders	Turkeys
NASEM [149]	3.6	2.1 ^4^	n/a	2.5–4.0
GfE [150]	2.9	2.5	2.5	n/a
Brazilian tables [151]	4.7–9.1	4.8 ^4^	8.0	n/a
FEDNA [152]	3.1–6.5	4.0	7.0	5.5–10.0
Cobb [153]	6.0–9.0	n/a	13.0	n/a
Aviagen [154,155] ^1^	7.0–9.0	n/a	10.0–16.0	4.0–10.0
Hubbard [156]	6.0–8.0	n/a	12	n/a
Hendrix (Hybrid turkeys) [157]	n/a	n/a	n/a	8.0–15.0
EW Group (laying hens) ^2^ [158,159,160]	n/a	4.0–6.6	n/a	n/a
Hendrix (laying hens) ^3^ [161,162,163,164]	n/a	5.0	n/a	n/a

^1^ Valid for Ross, Arbor Acres, and Indian River broiler breeds as well as Nicholas and B.U.T. medium and heavy turkey lines; ^2^ valid for Lohmann, Hy-Line, and H&N Nick layer breeds; ^3^ valid for ISA, Dekalb, Shaver, Bovans, Babcock, and Hisex layer breeds. ^4^ At 100 g of feed per hen daily. n/a = not applicable.

## Data Availability

Not applicable. No new data were created or analyzed in this study.

## Abbreviations

AMP	Adenosine monophosphate
ATP	Adenosine triphosphate
COX	Cytochrome c oxidase
ETC	Electron transport chain
FAD	Flavin adenine dinucleotide
FADH2	Dinucleotide adenine flavine molecule
FAO	The Food and Agriculture Organization of the United Nations
FCR	Feed conversion ratio
FMN	Flavin mononucleotide
G-6P-D	Glucose-6-phosphate dehydrogenase
GSH	Reduced form of glutathione
GSSG	Oxidized glutathione
Irg1	The immune responsive gene 1 protein
IUPAC	The International Union of Pure and Applied Chemistry
NADH	Dinucleotide nicotinamide molecules
NADPH	Nicotinamide adenine dinucleotide phosphate
Nox2	NADPH oxidase 2
PDH	Pyruvate dehydrogenase
Q	Coenzyme Q10
RBP	Riboflavin-binding protein
RF	Riboflavin
RFK	Riboflavin kinase
ROS	Reactive oxygen species
TCA	Tricarboxylic Acid cycle
TNF	Tumor necrosis factor
TNFR1	Tumor necrosis factor receptor 1
